# Green synthesis of silver iodide nanoparticles from *Kluyveromyces marxianus* M59 beta-glucan: characterization and anticancer activity

**DOI:** 10.1186/s12896-025-01046-5

**Published:** 2025-10-02

**Authors:** Berat Cinar-Acar

**Affiliations:** Department of Biology, Faculty of Science, Gazi University, Ankara, 906500 Türkiye

**Keywords:** *Kluyveromyces marxianus*, Beta-glucan, Silver iodide nanoparticles, Cytotoxicity, Green synthesis

## Abstract

Green synthesis of silver iodide nanoparticles (AgI-NPs) using carboxymethyl β-glucan from *Kluyveromyces marxianus* M59 was conducted to produce environmentally friendly and stable nanomaterials. The AgI-NPs had particle sizes ranging from 29 to 100 nm, exhibited a crystalline structure confirmed by X-ray diffraction, and and showed a characteristic absorption peak at 450 nm. Biological evaluations revealed low antibacterial activity against *Listeria monocytogenes* (maximum inhibition zone: 1.70 mm at 5 mg/mL), strong antibiofilm activity (66.9% reduction at 5 mg/mL), and significant antioxidant capacity, with the highest 2,2-diphenyl-1-picrylhydrazyl (DPPH) scavenging activity of 58.9% at 5 mg/mL. Cytotoxicity assays showed selective toxicity toward A549 lung cancer cells (IC50: 23.95 µg/mL at 24 h and 19.13 µg/mL at 48 h) compared to normal L929 fibroblasts (IC50: 130.37 µg/mL at 24 h and 73.69 µg/mL at 48 h). These findings highlight the multifunctional potential of AgI-NPs, emphasizing their antibiofilm, antioxidant, and anticancer activities, and provide a foundation for further investigations into their molecular mechanisms and sustainable applications.

## Introduction

Nanotechnology, the field of designing and controlling materials at the nanoscale, is rapidly expanding due to its numerous potential applications. Nanoparticles, defined as particles ranging in size from 1 to 100 nm, exhibit distinctive physical, chemical, and structural features [1–4]. Among various metal nanoparticles, silver nanoparticles (Ag NPs) stand out for for their antimicrobial, antioxidant and anticancer properties [5–8].

Nanomaterial synthesis can be classified into two primary categories: conventional and green methods [9]. While conventional techniques offer numerous advantages, such as producing a wide variety of nanoparticles with diverse applications, they often rely on hazardous organic solvents [10, 11], high temperatures, and energy-demanding processes. Moreover, these techniques contribute to environmental concerns, such as increased carbon dioxide emissions and the release of volatile vapors, that exacerbate the greenhouse effect. Therefore, this has led to a growing preference for green synthesis methods [12, 13].

In contrast, biosynthesis also referred to as green synthesis, offers several advantages in the nanoparticles production process such as avoiding toxic chemicals, lowering costs, minimizing environmental impact, and enabling the biological reduction and stabilization of metal ions [14–16]. Biologically active compounds can be extracted from microorganisms (bacteria, fungi, yeast, algae), plants, and animals [17]. Silver nanoparticles synthesized using these biological materials are known as green nanoparticles [18, 19]. The antimicrobial activity of Ag NPs is primarily attributed to the release of silver ions and the generation of Reactive Oxygen Species (ROS), which disrupt key cellular functions such as nucleic acid and protein synthesis, membrane integrity, and mitochondrial activity. These disruptions ultimately result in microbial cell death. Additionally, Ag NPs may exert direct bactericidal effects [2, 12, 20].

Green synthesis of silver nanoparticles (Ag NPs) is favored for its strong antimicrobial activity, low toxicity, and high thermal stability, supporting its use in consumer products and medical applications. Green synthesis of Ag NPs has surpassed other nanoparticles synthesis methods in popularity due to their potent antimicrobial properties with minimal toxicity [21] and exceptional thermal stability [22]. These attributes have led to their incorporation in the production of everyday items like textiles, plastics, electronic devices, and cosmetics, as well as in cancer therapy and advanced drug delivery systems [23].

Although Ag NPs have been widely investigated for their broad-spectrum antimicrobial activity and physicochemical versatility, silver iodide nanoparticles (AgI-NPs) have recently garnered attention due to their distinct physicobiological properties. Notably, AgI-NPs exhibit enhanced photocatalytic efficiency under visible light irradiation, attributed to their relatively narrow bandgap energy (~ 2.8 eV), which facilitates ROS generation a key mechanism underlying microbial inactivation and biomolecule degradation. Furthermore, AgI-NPs demonstrate superior photostability and biocompatibility compared to Ag NPs, rendering them particularly suitable for applications in biomedical fields, including antimicrobial coatings, wound healing, and drug delivery systems. Silver iodide is a versatile material with diverse applications across various industries. Its unique properties make it valuable in photography, medicine, electronics, magnetics, optics, and catalysis for metals and semiconductors. Additionally, silver iodide nanoparticles have demonstrated antibacterial potential, providing a potential means to combat microbial infections [24, 25].

Microorganisms, particularly yeasts, act as eco-friendly nanofactories for the intra- and extracellular synthesis of metal nanoparticles, utilizing metabolites such as proteins, carbohydrates, and phenolic compounds [26]. Yeasts are preferred over bacteria for nanoparticles production due to their ease of cultivation, rapid growth, and scalability in simple media [27, 28]. Yeasts are single-cell eukaryotic fungi with broad applications across biotechnology, medicine, and environmental sciences. Among the industrially relevant species, *Kluyveromyces marxianus* has gained attention due to its GRAS status, rapid growth, thermotolerance, and ability to utilize diverse carbon sources [29–32]. Although limited genetic and physiological knowledge initially restricted its use, recent advances in fermentation and growth optimization have enhanced its applicability, particularly in the production of biofuels and value-added compounds in food and pharmaceutical industries [33–35].

Beta-glucans (β-glucans) are polysaccharides commonly found in the cell walls of fungi, yeast, bacteria, and grains [36, 37]. As a main component of the yeast cell wall, β-glucans, along with mannoproteins, are among the most functionally active biomolecules. Biologically, β-glucans play a key role in cell wall integrity, immune modulation, and protection against environmental stress in microorganisms [38]. β-glucan derived from yeast contributes to the host’s defense against infection by exhibiting antibacterial properties against bacteria and pathogenic microorganisms [39]. Glucose polymers, linked by β-1,3 and β-1,6 glycosidic bonds, offer versatile functionalities in the food industry, acting as emulsifiers, stabilizers, and thickeners [40]. Beyond their food applications, β-glucans have shown potential health benefits, including disease diagnosis and treatment, anti-inflammatory effects, immune modulation, cancer, wound-healing, metabolism regulation, and gut health support [41, 42]. The prebiotic potential of β-glucans and their expanding applications in biotechnology highlight their significance in various fields [38, 43].

This study explores the synthesis of AgI-NP using C-β-glucan extracted from *Kluyveromyces marxianus* M59 rather than directly utilizing the microorganism, as β-glucan is a well-characterized polysaccharide with a defined chemical structure and functional groups (e.g., hydroxyl groups), allowing for a more controlled, consistent, and reproducible nanoparticles synthesis process. The synthesized AgI-NPs were characterized (SEM, FT-IR, XRD) and evaluated for their antibacterial, antibiofilm, antioxidant properties. Additionally, the cytotoxicity of AgI-NPs was assessed in both normal and cancer cells. Given the absence of studies synthesizing silver nanoparticles from the cell wall beta-glucan of *K. marxianus* and characterizing and determining the biological activity of the synthesized nanoparticles, this study aims to fill this gap in the literature and guide future studies.

## Materials and methods

The *K. marxianus* M59 strain, isolated from tulum cheese and molecularly identified using 18 S RNA sequencing (GenBank Accession No: KP132323.1), was obtained from the Gazi University Department of Biology Biotechnology Laboratory culture collection [44]. Yeast-Peptone-Dextrose, (YPD, Merck) medium was used to cultivate the yeast, and pre-activation was performed by inoculating 2% samples into the liquid medium and incubating at 37 °C for 24–48 h with agitation at 150 rpm.

### β-glucan extraction

**Fig. 1:**
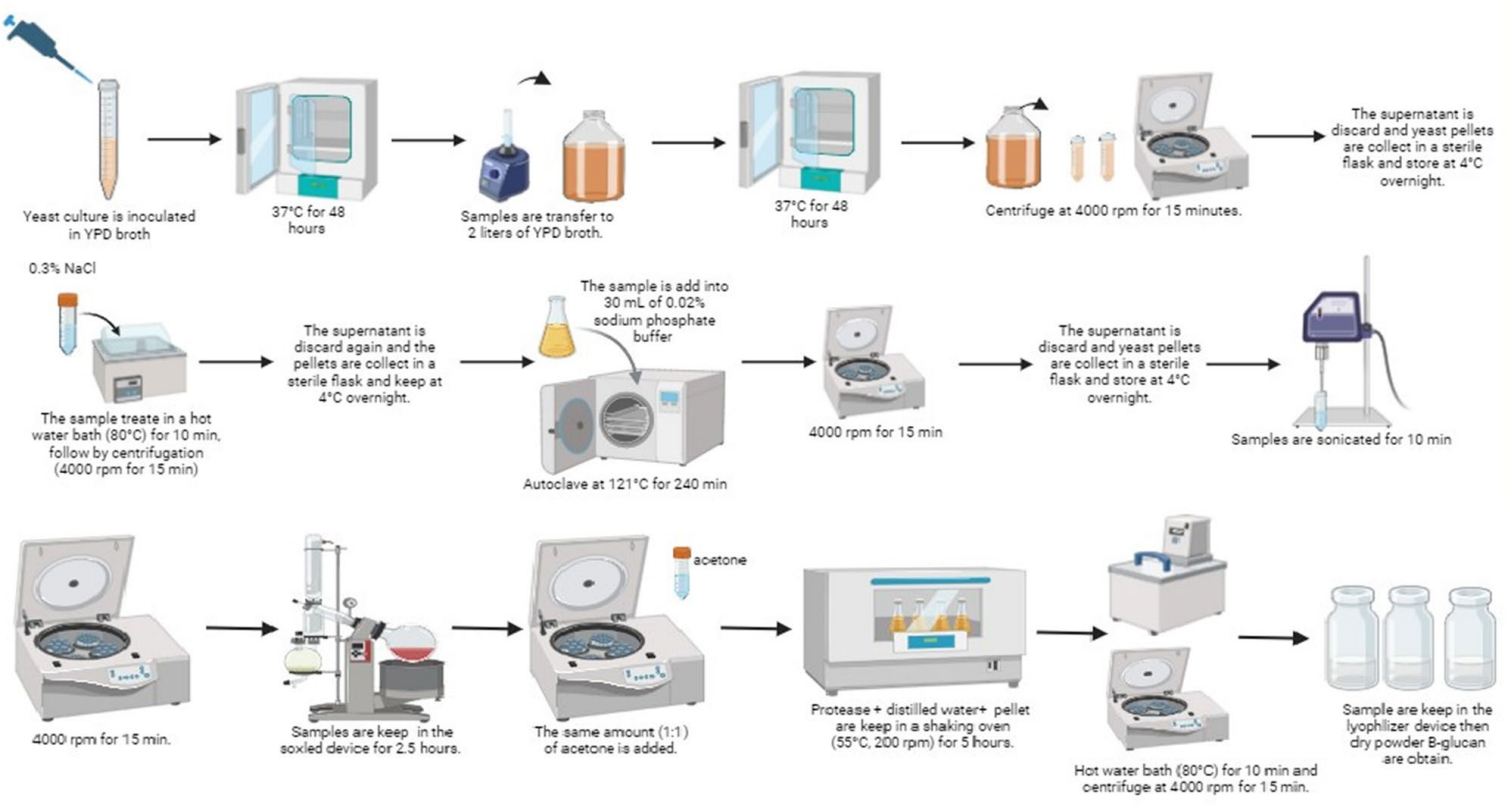
β-glucan extraction (Drawn with BioRender)

β-glucan (β-gluM59) composed of β-(1→3) linked glucose units and β-(1→6) linked branches was extracted from the cell wall of the *K. marxianus* M59 yeast strain, using Liu et al. [45] and Magnani et al. [46] methods. The *K. marxianus* M59 yeast pellet was treated with 30 mL of sterile 3% NaCl and kept in a shaking oven at 200 rpm, 55 °C, for 24 h. The supernatant was discarded, and the sample was stored at + 4 °C. The autolysate was heat-treated at 80 °C for 10 min, centrifuged (4000 rpm, 15 min), and stored at + 4 °C. To further disrupt the cell walls and release intracellular components, the autolysate was treated with 30 mL of 0.02% sodium phosphate buffer and glass beads. The mixture was then autoclaved at 121 °C for 240 min. After autoclaving, supernatant was discarded, the remaining pellet kept overnight. The sample was sonicated (20 kHz, 10 min) in an ice bath with 25 mL distilled water and then treated with isopropanol in a Soxhlet extractor for 2.5 h to remove lipids. The remaining residue was weighed and washed twice with acetone at a ratio of 1 mL per gram of sample (1:1, w/v), followed by a single wash with distilled water. After these washing steps, the sample was centrifuged. To eliminate proteins from the sample, 0.4 units of protease enzyme (Sigma) and 10 milliliters of distilled water were added. The enzyme was activated by incubating the mixture at 55 °C for a duration of 5 h. The sample was placed in a hot water bath at 80 °C for 10 min to deactivate the enzyme [47]. The pellet was centrifuged and dissolved in 5 mL water. The solution was put into the vials and stored at -80 °C stored. Subsequently, the sample underwent lyophilization using a Christ alpha 2–4 LD Plus milling device. The extraction steps are illustrated in Fig. 1.

### Carboxymethylation

Carboxymethylation, a chemical modification technique, was employed to enhance the solubility of β-gluM59. 90 mg biopolymer was treated with 5.4 mL isopropanol until homogenized. Then, 30% NaOH (10 mL) was added. The magnetic stirrer was set to 50 °C and 1.1 g of monochloroacetic acid (MCA) was put into the mixture and stirred for 6.5 h. The mixture was cooled, adjusted to pH 7.0, and neutralized with 96% alcohol in a 1:1, v/v ratio [48–51]. After de-alcoholization with an evaporator (Heidolph Laborota 4000 efficient Rotary Evaporator), the mixture was lyophilized to obtain a dry, water-soluble powder of carboxymethylated β-glucan (C-β-glucan).

### Silver iodide nanoparticles synthesis from C-β-glucan-M59

**Fig. 2:**
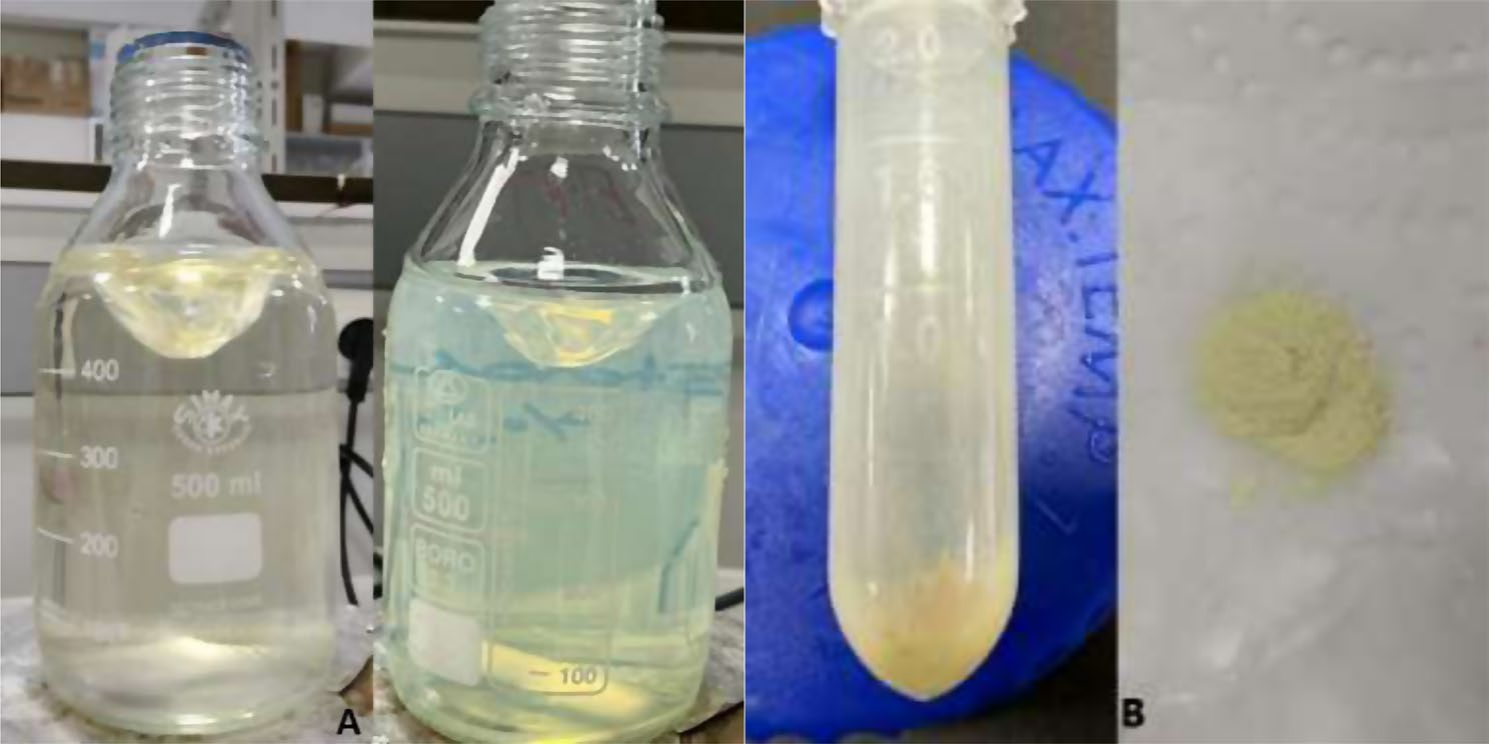
(**a**-**b**) Visual monitoring of AgI-NP synthesis **a**) Color change (yellow) observed in the silver solution, **b**) Synthesized iodide silver nanoparticles

Silver nanoparticles were synthesized from C-β-glucan (pH 7.0) using a method adapted from Sun et al. [52]. A 1:1 (v/v) mixture of silver nitrate (3 mM) and potassium iodide (30 mM) was added to a C-β-glucan (1 mg/mL) solution. The resulting mixture was stirred at 150 rpm for 30 min, and then centrifuged at 13,000 rpm for 15 min. The successful biosynthesis of AgI-NPs was indicated by a distinct yellow color change, which is attributed to surface plasmon resonance (SPR) resulting from the formation of silver iodide nanoparticles (Fig. 2a). The supernatant was discarded, and the nanoparticles pellet was collected and washed thoroughly with deionized water to remove unreacted reagents and impurities. Sonication (20 kHz, 5 min) was applied to disperse the nanoparticles uniformly before drying (Fig. 2b). To prevent oxidation, the sample was stored in an opaque container, shielded from light, and subsequently utilized.

### UV-Vis spectroscopy analysis of AgI-NPs

The AgI-NPs were diluted with deionized water, and transferred to a quartz cuvette. The samples were then scanned using a UV-Vis spectrophotometer (Hitachi, U-1800) in the wavelength range of 200–800 nm.

### Characterization studies of AgI-NPs

#### Scanning electron microscopy (SEM) characterization

SEM was employed to investigate the morphological characteristics of the synthesized silver nanoparticles. Before SEM analysis, the nanoparticles samples were resuspended in deionized water. For fixation, 2.5% glutaraldehyde prepared in 0.2 M phosphate-buffered saline (PBS, pH 7.2) was added to the nanoparticles solution. After incubating at + 4 °C for 2 h, the sample was centrifuged again. The supernatant was discarded, and the nanoparticles were resuspended in PBS. The nanoparticles were washed twice more with PBS, followed by dehydration through a graded ethanol series (50%, 60%, 70%, 80%, 95%, and 99.5%). The sample was then transferred to amyl acetate. Afterward, the sample was dried using the critical-point drying method with CO₂ (Polaron, CPD 7501) and coated with gold using a Polaron SC 502 sputter coater.

The morphology and size of the AgI-NPs were analyzed using Scanning Electron Microscopy (SEM) in combination with Energy Dispersive X-ray Spectroscopy (EDS) for elemental analysis and mapping (QUANTA 400 F Field Emission, FEI Company, Hillsboro, Oregon, USA), which operates at a resolution of 1.2 nm. The microstructure of thin films containing AgI-NPs was further examined with high-precision using the FEI Tecnai G2 Spirit BioTwin Transmission Electron Microscope (TEM) in high-vacuum mode, with an accelerating voltage range of 20 to 120 kV. SEM analysis was conducted at the ODTU Materials Research Laboratory (MERLAB) in Ankara, Turkey.

#### Fourier transform infrared (FT-IR) spectrometer analysis

FT-IR analysis was used to identify the functional groups on the nanoparticles surface. This technique provides important insights into the chemical bonds present in the nanoparticle molecules or their composite structures. A concentrated nanoparticles solution was dried to obtain a powdered sample, a small amount of which was placed onto the sample holder for FT-IR analysis. The spectra were recorded using a Bruker IFS 66/S FT-IR spectrometer equipped with a Hyperion 1000 microscope at METU MERLAB.

#### X-ray diffraction (XRD) analysis

XRD analysis was performed to designate the structure of the nanoparticles using a Rigaku MiniFlex Desktop X-ray Diffractometer. The operating parameters were set at 40 kV and 15 mA, with a scan range of 3° to 90° 2θ using CuKα radiation (wavelength λ = 1.5406 Å). The software used for phase identification is PDXL, and the database employed consists of approximately 200,000 entries from ICDD cards. Analysis was conducted at ODTU MERLAB.

### Biological activity

#### Antibacterial activity

The antibacterial activity of AgI-NPs against Gram (+) *S. aureus* ATCC 25,923, *L. monocytogenes* ATCC 7644, and Gram (-) bacteria *P. aeruginosa* ATCC 29,212, *E. coli* O157:H7 was evaluated using the agar well diffusion method. The test bacteria were grown in Nutrient Broth at 37 °C for 24 h. Following incubation, the bacterial cultures were centrifuged and resuspended in sterile 0.85% NaCl solution, and the turbidity was adjusted to 0.5 McFarland standard (1.5 × 10⁸ CFU/mL), From each standardized suspension, 100 µL was spread evenly onto sterile Nutrient Agar plates using a sterile swab. Wells of 6 mm diameter were punched into the agar, and 100 µL of AgI-NP suspensions at concentrations of 1, 3, and 5 mg/mL were loaded into each well. The plates were incubated at 37 °C for 24 h. All experiments were performed in duplicate. Standard ampicillin antibiotic disks (10 µg) were used as positive controls. After incubation, the diameters of the inhibition zones were measured in millimeters using a using a digital vernier caliper [53].

#### Antibiofilm activity

The antibiofilm activities of AgI-NPs against *S. aureus*,* L. monocytogenes*,* P. aeruginosa*, and *E. coli* were determined. Ag NPs nanoparticles were prepared at concentrations of 1, 3, and 5 mg/mL and sonicated for 5 min. 20 µL of the homogenized samples were transferred to well plates containing 180 µL of bacterial culture (McFarland 0.5). The control group consisted of two conditions: the well containing only the growth medium (negative control) and the well containing both the growth medium and the test bacteria (positive control). The plates were incubated at 37 °C for 24 h, then washed with PBS (0,01 mol/L KH_2_PO_4_/K_2_HPO_4_ and 0,15 mol/L NaCI, pH: 7,0) to remove planktonic cells. After drying, 150 µL of 99% methanol (Merck) was added to each well at room condition and kept for 20 min. The wells were washed with 150 µL of 1% crystal violet solution (Merck), incubated for 15 min, and washed again with tap water. 150 µL of 33% glacial acetic acid was added, kept for 15 min. The plate was measured using an ELISA reader (Epoc, BioTek) at 570 nm [54].

$$\begin{aligned}\:&\%\:Biofilm\:Inhibition\\&=\left[\right({\text{O}\text{D}}_{\text{C}\text{o}\text{n}\text{t}\text{r}\text{o}\text{l}}-\:{\text{O}\text{D}}_{\text{S}\text{a}\text{m}\text{p}\text{l}\text{e}})\:/\:{\text{O}\text{D}}_{\text{C}\text{o}\text{n}\text{t}\text{r}\text{o}\text{l}}]\:\text{x}\:100\:\end{aligned}$$

A_Sample_: The absorbance of the sample.

A_Control_: The absorbance of the control.

#### Antioxidant activity

The antioxidant activity of AgI-NPs was assessed using 2,2-diphenyl-1-picrylhydrazyl (DPPH), superoxide anion, and hydroxyl radical scavenging assays [55–59]. Ascorbic acid (Sigma-Aldrich, ≥ 99%) was used as a control. Ascorbic acid was dissolved in distilled water to a final concentration of 1 mg/mL.

##### DPPH radical scavenging effect

One milliliter of AgI-NP suspension (1, 3, and 5 mg/mL) was mixed with 1 mL of DPPH solution (0.008% w/v in methanol) waited in the dark for 30 min. The absorbance was then measured at 517 nm.

$$\begin{aligned}\:&\text{A}\text{n}\text{t}\text{i}\text{o}\text{x}\text{i}\text{d}\text{a}\text{n}\text{t}\:\text{a}\text{c}\text{t}\text{i}\text{v}\text{i}\text{t}\text{y}\:\left(\text{\%}\right)\\&=[1-(\text{B}517\:/\:\text{C}517\left)\right]\:\times\:\:100\text{\%}\end{aligned}$$

B_517_: The absorbance of nanoparticles treated with DPPH.

C_517_: The absorbance of control group (DPPH solution).

##### Superoxide anion activity

0.2 mL of pyrogallol (3 mM) was added to AgI-NP solutions (1, 3, and 5 mg/mL) and kept for 5 min. The reaction was stopped with 10 mM HCl, and the absorbance was measured at 325 nm.

$$\begin{aligned}\:&\text{\%}\:\text{S}\text{u}\text{p}\text{e}\text{r}\text{o}\text{x}\text{i}\text{d}\text{e}\:\text{a}\text{n}\text{i}\text{o}\text{n}\:\text{a}\text{c}\text{t}\text{i}\text{v}\text{i}\text{t}\text{y}\\&=\left[\right(\text{A}0\:-\:\text{A}1)\:/\:\text{A}0]\:\text{x}\:100\end{aligned}$$

A_1_: Absorbance value of samples.

A_0_: Absorbance value of the solution without sample.

##### Hydroxyl radical scavenging activity

One milliliter (mL) of brilliant blue (0.435 mM), 2 mL of FeSO_4_ (0.5 mM), and 1.5 mL of H_2_O_2_ (3% w/v) were mixed with Ag NPs samples and kept at 37 °C for 1 h. The absorbance was then measured at 624 nm.

$$\begin{aligned}\:&\text{\%}\:\text{H}\text{y}\text{d}\text{r}\text{o}\text{x}\text{y}\text{l}\:\text{r}\text{a}\text{d}\text{i}\text{c}\text{a}\text{l}\:\text{s}\text{c}\text{a}\text{v}\text{e}\text{n}\text{g}\text{i}\text{n}\text{g}\:\text{a}\text{c}\text{t}\text{i}\text{v}\text{i}\text{t}\text{y}\\&=\left[\right(\text{A}0-\:\text{A}1)/\:(\text{A}-\:\text{A}1\left)\right]\:\text{x}\:100\text{\%}\end{aligned}$$

A_0_: Solution containing the sample at a certain concentration.

A_1_: Solution in the absence of sample.

A: Sample and the solution not containing the Fenton reaction system.

### Cytotoxicity effect of AgI-NP against A549 and L929 cells

The cytotoxic effects of AgI-NPs on L929 normal and A549 human lung cancer cells were investigated in this study. Cells were exposed to various concentrations (12.5, 25, 50, and 100 µg/mL) of the nanoparticles for 24 and 48 h. Cell viability was assessed using the MTT assay [60, 61]. The analysis was conducted using services provided by Eskisehir Osmangazi University Central Research Laboratory Application and Research Center. While specific conditions (e.g., cell passage, confluency, volumes) were optimized by the laboratory, standard protocols for cytotoxicity testing were followed. Additionally, all materials, reagents, and equipment used in the assays were sterilized according to the laboratory’s established procedures to prevent contamination and ensure reliable results.

A549 cells were cultured in DMEM supplemented with 10% fetal bovine serum (FBS) and monitored until ~ 70% confluency was achieved. The cells were then detached using trypsin-EDTA, centrifuged, and seeded into 96-well plates at a density of 1 × 10⁴ cells/well. AgI-NPs were initially prepared at a concentration of 1 mg/mL, exposed to UV light for 30 min, and homogenized in the culture medium using a shaker for 24 h. The working concentrations were obtained by diluting this stock solution in complete medium.

Once the seeded cells reached approximately 70% confluency, the culture medium was replaced with fresh medium containing the test samples. Negative control wells contained only cells and medium (untreated control), while positive control wells were treated with 2 µL of hydrogen peroxide (H₂O₂), a known cytotoxic agent. Blank wells containing only medium (without cells) were used for background correction.

After 22 h of incubation, 20 µL of WST-8 solution (10% of the total well volume) was added to each well. The plates were wrapped in aluminum foil to protect them from light and incubated for an additional 2 h. Absorbance was then measured at 450 nm with a reference wavelength of 630 nm using a microplate reader.

Cell viability was calculated using the following equation:

$$\begin{aligned}\:&\text{C}\text{e}\text{l}\text{l}\:\text{v}\text{i}\text{a}\text{b}\text{i}\text{l}\text{i}\text{t}\text{y}\left(\text{\%}\right)\\&=(\text{A}\text{v}\text{e}\text{r}\text{a}\text{g}\text{e}\:\text{a}\text{b}\text{s}\text{o}\text{r}\text{b}\text{a}\text{n}\text{c}\text{e}\:\text{o}\text{f}\:\text{t}\text{r}\text{e}\text{a}\text{t}\text{e}\text{d}\\&\quad/\text{A}\text{v}\text{e}\text{r}\text{a}\text{g}\text{e}\:\text{a}\text{b}\text{s}\text{o}\text{r}\text{b}\text{a}\text{n}\text{c}\text{e}\:\text{o}\text{f}\:\text{c}\text{o}\text{n}\text{t}\text{r}\text{o}\text{l}\:\text{g}\text{r}\text{o}\text{u}\text{p})\\&\quad\text{x}\:100\end{aligned}$$

The cytotoxic effects of the synthesized AgI nanoparticles were evaluated using ISO 10993-5 standards (50% or more: high cytotoxic, 21–50%: moderate, 11–20%: low, and 10% or less: non-cytotoxic). The results were used to determine the IC50 value.

### Statistical analysis

All studies were performed in triplicate, and the average results are presented as mean ± standard deviation. The statistical analyses (One-way ANOVA/Tukey tests and Pearson correlation) were carried out using SPSS Inc. Software 22.0.

## Results and discussion

Due to the limited research on silver iodide nanoparticles synthesis, the experimental results were compared to those obtained from studies on silver nanoparticles.

### UV-Vis spectrum of AgI-NPs

**Fig. 3:**
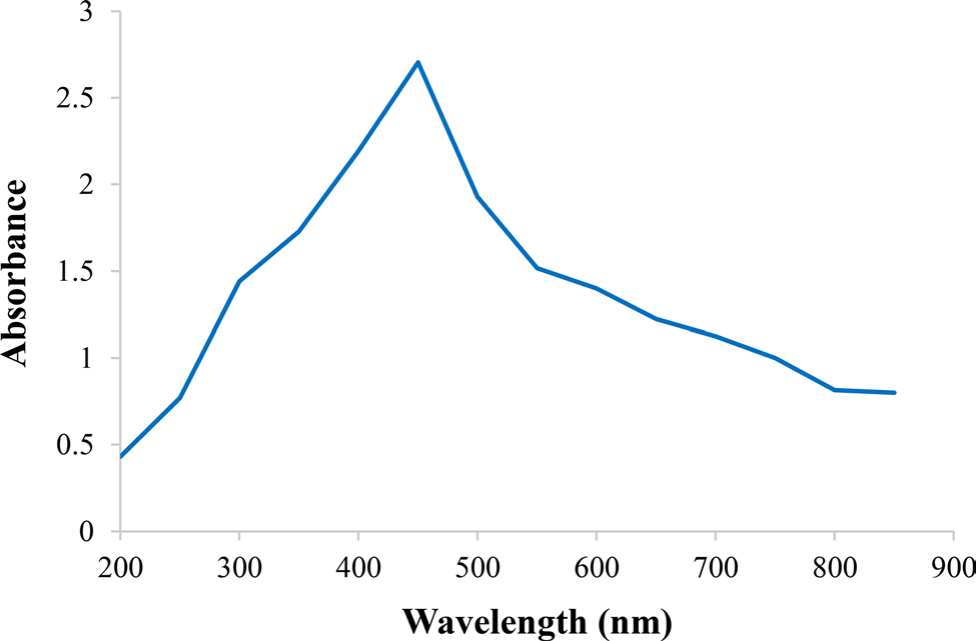
UV-Vis spectrum of AgI-NP

The successful synthesis of AgI-NPs from C-β-glucan was indicated by the transformation of the solution into a yellow color (Fig. 2a). Upon scanning the nanoparticles within the wavelength range of 200 to 800 nanometers, an absorption peak characteristic of AgI-NPs was observed at 450 nanometers (Fig. 3).

The AgI-NPs in the Feyadh and Mohammed [62] study were produced through a chemical reduction process. Their absorbance spectra were found to peak at a wavelength of 425 nanometers. Alwhibi et al. [63] reported a slightly higher peak at 450 nm. In another study, the maximum absorbance peak was observed at 427.81 nm [64]. Yuksekdag et al. [61] reported that biogenic Ag NPs synthesized from postbiotics exhibited an absorption peak at 435 nm. In contrast, Abu-Hussien et al. [65] observed an absorption peak at 455 nm for similar AgNPs.

The UV–visible spectrum of the synthesized AgI-NPs showed a distinct absorption peak at 450 nm, confirming nanoparticles formation in this study. When compared with previous studies, the absorbance peaks reported for AgI or Ag-based nanoparticles varied slightly, generally ranging from 415 to 455 nm. These slight variations in absorption peaks may be attributed to differences in synthesis methods, particle size, and stabilizing agents. Overall, the UV–Vis spectral data obtained in this study are in line with previous reports, supporting the successful formation of AgI-NPs.

### Scanning electron microscopy (SEM) characterization

**Fig. 4:**
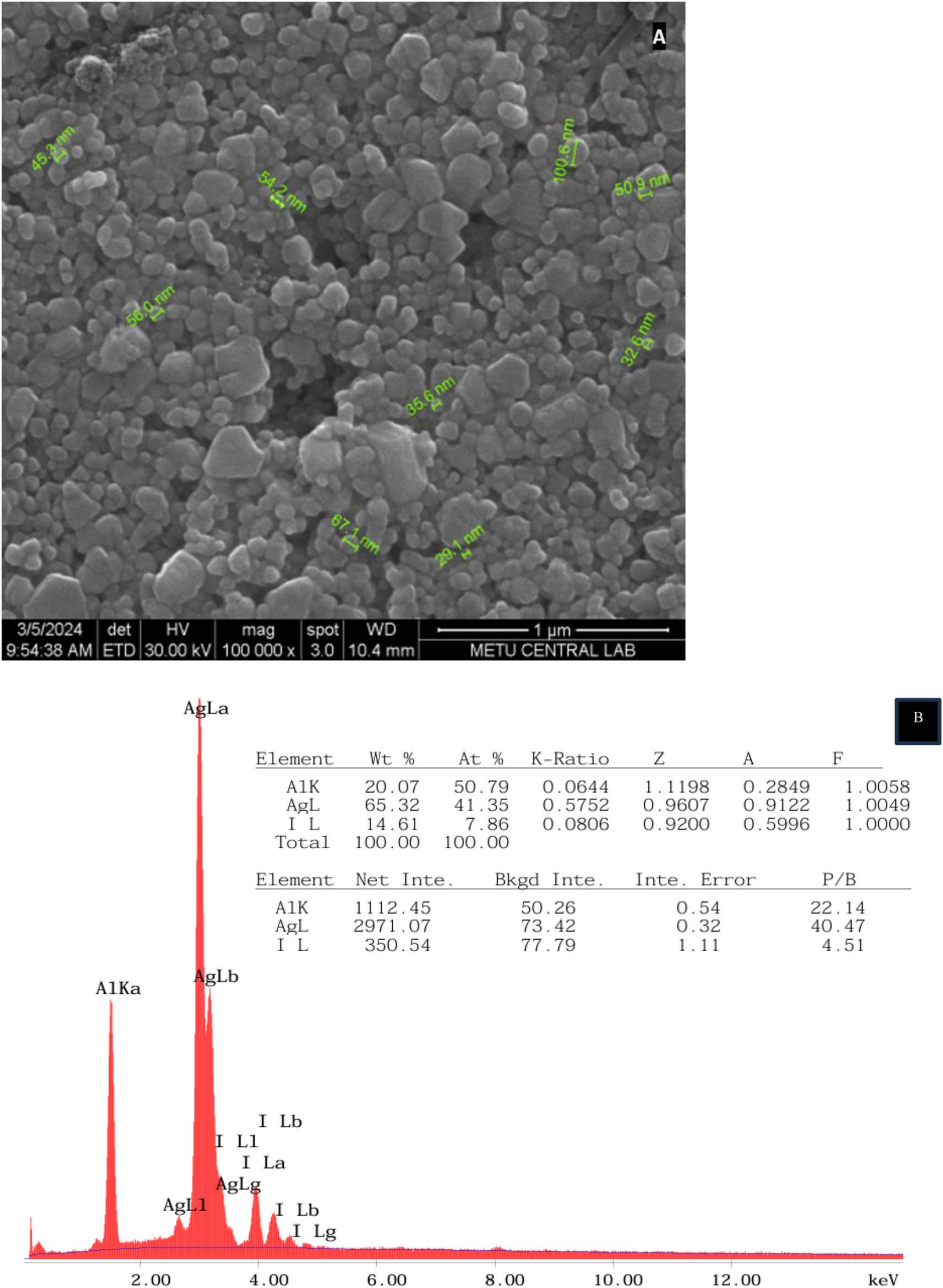
SEM photographs (**a**) and EDX spectrum (**b**) of AgI-NP

AgI-NPs displayed a spherical morphology with diameters ranging from 29.1 to 100.6 nm (mean diameter 52.6 ± 20.5) (Fig. 4a). This result shows that spherical AgI nanoparticles were obtained from AgNO_3_ and KI. EDX analysis of the biosynthesized AgI-NPs revealed peaks for Ag, I, and Al (Fig. 4b). EDX analysis indicated the presence of silver constituting 65.32% and iodine (14.61%) of the total weight.

In general, the findings of this study align with previous studies in terms of nanoparticle size and elemental composition (Table 1). However, the detection of aluminum and the significant oxygen content suggest slight variations in the synthesis and/or sample preparation processes. These differences, however, are not unusual and may reflect the specific conditions under which the nanoparticles were synthesized and analyzed.

**Table 1:** Morphological and compositional characteristics of agI-NPs and ag NPs

Morphology	Size (nm)	EDX composition (%by weight)	Synthesis methods	Reference
Semi-spherical	22–73	Ag: 51.5, I: 48.5	Chemically synthesized, possible agglomeration	[62]
Not specified	45–50	Not specified	Chemically synthesized AgI- NPs	[66]
Spherical	40.19	Ag: 82.4, Au, C, O, Cl: Not specified	Ag NPs from *C. albicans* supernatant	[67]
Spherical, smooth	20–50	Not specified	*β*-d-glucanNPsfrom*P. aphanidermatum*	[68]
Spherical	*β*GNPs:58.65, Ag NPs:6.72, *Β*GAg NPs: 63.88	Not specified	Yeast *β*-glucan, negative surface charge	[69]
Not specified	11–25	Ag: High purity, C, Cu, Mg, Si: Trace	Ag NPs, high purity, TEM grid impurities	[70]
Spherical	29.1–100.6	Ag: 65.32, I: 14.61, Al: Not specified	Biosynthesized from AgNO_3_ and KI	This study

### Fourier transform infrared (FT-IR) spectrometer analysis

**Fig. 5:**
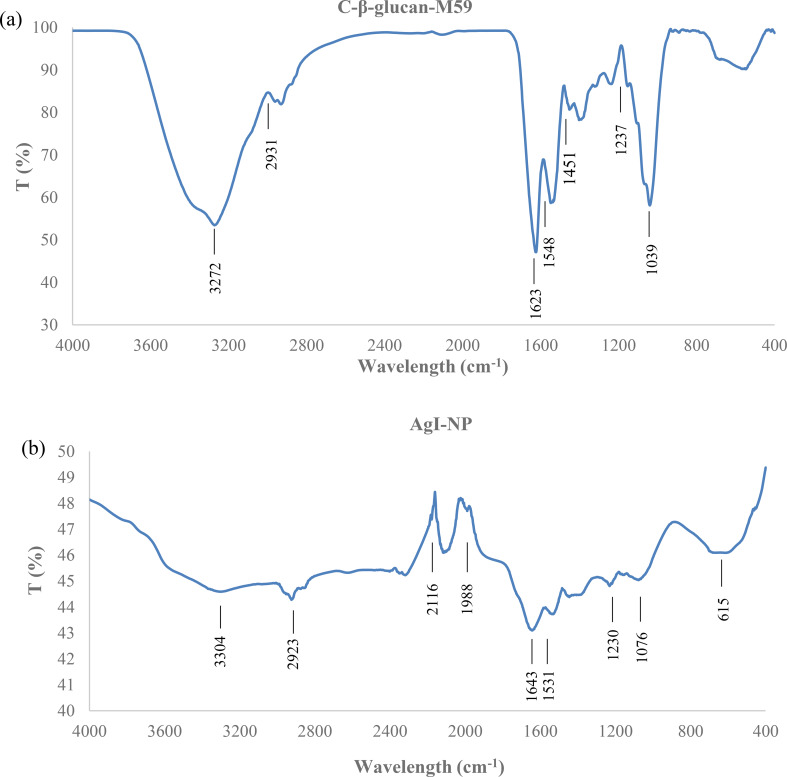
FT-IR spectrum of C-β-glucan-M59 (**a**) and AgI-NP (**b**)

The FTIR spectrum of beta-glucan reveals distinct absorption bands that confirm its polysaccharide structure. A broad peak at 3300–3400 cm⁻¹ indicates O-H stretching from hydroxyl groups, while a band at 2900 cm⁻¹ reflects C-H stretching. Peaks between 1000 and 1200 cm⁻¹, notably at 1028 cm⁻¹ and 1076 cm⁻¹, correspond to C-O-C and C-O stretching, confirming glycosidic linkages and pyranose ring structures. A minor absorption at 1600–1650 cm⁻¹ indicates carboxymethylation (Fig. 5a).

The FT-IR spectra of the synthesized AgI-NPs revealed a peak at 3304 cm^− 1^ attributable to the stretching vibration of hydroxyl groups (–OH) [69, 71]. The peak at 2923 cm^− 1^ corresponds to the C-H stretch [72, 73], while C-C stretching is observed at 1643 cm^− 1^ [71, 74]. The 1531 cm^− 1^ peak is stretching of the C = O bond [75, 76], the characteristic peaks at 1230 cm⁻¹ and 1076 cm⁻¹, suggesting the presence of O-C stretching vibrations [76]. The peak found at 615 cm⁻¹ can be attributed to the presence of Iodo-compound (C-I) stretching (Fig. 5b).

The FT-IR spectra of the synthesized AgI-NPs in this study exhibited characteristic peaks similar to those observed in previous research (Table 2). While the results align with existing literature, some variations in peak intensities and positions may be attributed to differences in synthesis methods or sample preparation conditions.

**Table 2:** Comparison of FT-IR characteristic peaks for AgI-NPs and related nanoparticles from various studies

Peak (cm − 1)	Functional group	Chemical assignment	Reference
3439.29, 1619.72	O-H stretching, bending	Alcohol groups	[62]
3932.8, 3786.75	O-H stretching	Hydroxyl groups	
2925.38	C-H stretching	SDS coating	
1385.19	N = O stretching	Nitro groups	
615.45	C-I stretching	Iodo-compound	
1024.39	C-N stretching	Amines	
3420	O-H stretching	Carboxymethyl beta-glucan	[77]
2800–2900, 1400	CH_3_ vibrations	Methyl groups	
1715, 1098	C = O, C-O vibrations	Carbonyl groups	
1600	C = C vibrations	Conjugated carbon	
3367, 3422	O-H stretching	*β*G-NPs, *β*G-Ag NPs	[69]
2914, 2929	C-H stretching	Aliphatic groups	
1721, 1099, 1029	C-O, O-H stretching	Carbon-oxygen bonds	
3419, 3321, 3417	O-H stretching	Hydroxyl groups	[37]
2922, 2933, 1375	C-H stretching	Free hydroxyl/carboxyl	
1031, 1020, 1111	C-O-C stretching	*β*-glucan bonds	
3167, 3227	O-H stretching	Hydroxyl groups	[61]
2922, 2974	C-H stretching	Aliphatic groups	
1637, 1526, 1581	Amide I, II bands	Protein/peptide bonds	
1212, 1236, 1052	C-N, C = O stretching	Amine/carboxyl groups	
3753 3464 2926 1644 1388 1240, 1126, 1055 827, 613, 500	O-H stretching OH stretching C-H stretching O-H bendin N = O stretching C-N stretching C-I stretching	Hydroxyl group Alcohol groups SDS coating Alcohol groups Nitro groups Amines Iodo-compounds	[65]
3490 3398, 3365, 3336, 3301, 3284, 3176, 3114 2356 1220, 1137, 106 628, 594	O-H stretching N-H stretching C ≡ N stretching C-N stretching C-I stretching	Alcohol or phenol Amines, amides, or related compounds Amines or amides Sulfur-containing groups	[78]
3304	O-H stretching	Hydroxyl groups	This study
2923	C-H stretching	Aliphatic C-H	
1643	C-C stretching	Carbon-carbon bonds	
1531	C = O stretching	Carbonyl groups	
1230, 1076	O-C stretching	Oxygen-carbon bonds	
615	C-I stretching	Iodo-compound	

### X-ray diffraction (XRD) analysis

**Fig. 6:**
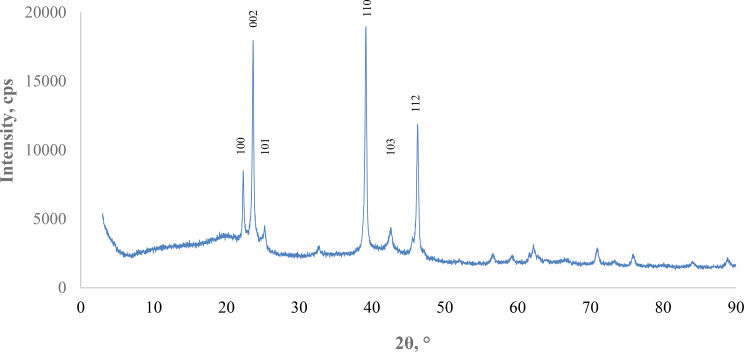
X-ray diffraction spectra of AgI-NP

In the XRD pattern, 2θ diffraction peaks were observed at 22.32°, 23.68°, 25.29°, 39.15°, 42.62°, and 46.29°. It was determined that these peaks correspond to the reflection plane of AgI-NPs indices (100), (002), (101), (110), (103), (112), respectively. According to these results, it was determined that AgI-NPs have a face-centered cubic (FCC) crystal structure (Fig. 6).

In a separate study, XRD analysis of AgI-NPs revealed diffraction peaks at 22.49°, 23.77°, 25.35°, 33.02°, 39.31°, 42.82°, 46.28°, 59.59°, 62.26°, 71.40°, and 76.08°. These peaks matched the reflection planes of AgI-NPs indices (100), (002), (101), (102), (110), (103), (112), (203), (210), (300), and (213), respectively [62]. Ismail et al. [79] reported that AgI NPs synthesized by laser ablation exhibited characteristic diffraction peaks at 22.1°, 24.36°, 34.34°, 39.52°, 42.6°, and 45.14° corresponding to the (100), (002), (101), (102), (110), (112), and (103) crystallographic planes. Kaliammal et al. [80] studied polymer-protected AgI-NPs. Their XRD analysis revealed diffraction peaks at 23.9°, indicative of the β form of silver iodide, and at 39.2° and 46.4°, characteristic of the γ form of silver iodide.

The XRD pattern of the synthesized Ag NPs, as reported by Elnagar et al. [69], exhibited four prominent diffraction peaks at 2θ angles of 37.4°, 44.1°, 64.5°, and 77.3°, corresponding to the (111), (200), (220), and (311) crystallographic planes of face-centered cubic silver, respectively. Salem [81] investigated the biosynthesis of Ag NPs using *Saccharomyces cerevisiae*. XRD analysis confirmed a monoclinic structure of the Ag NPs, characterized by diffraction peaks at 2θ angles of 38.2° (100), 44.2° (200), 64°[68 (220), and 77° (311), indicative of the Ag crystal lattice. The Ag NPs synthesis by Kthiri et al. [70] via a green biosynthesis approach using *S. cerevisiae* exhibited a well-defined FCC lattice structure. This was confirmed by XRD analysis, revealing sharp diffraction peaks at 38.28°, 44.33°, and 64.33°, which are characteristic of the (111), (200), and (220) planes of metallic silver, respectively.

The XRD pattern of the synthesized AgI-NPs in this study displayed diffraction peaks corresponding to expected reflection planes, confirming their FCC crystal structure. These results are generally consistent with previous studies, though slight differences in peak positions and intensities can be attributed to variations in synthesis methods and sample preparation conditions. Such discrepancies are typical and reflect the inherent differences in nanoparticles production techniques and experimental conditions.

### Antibacterial activity

The antibacterial mechanisms of silver nanoparticles (Ag NPs) remain under investigation, with multiple pathways proposed, including: *(i) generation of reactive oxygen species (ROS)*,* (ii) disruption of bacterial cell walls and cytoplasmic membranes*,* (iii) inhibition of ribosomal function*,* and (iv) release of silver ions (Ag*⁺*) through oxidative processes* [61, 82, 83].

AgNPs exert their antibacterial effects through a multifaceted mechanism that compromises bacterial cell integrity and function. Initially, they interact with the cell wall, causing structural damage and increasing membrane permeability. This disturbance leads to loss of intracellular homeostasis and leakage of cellular contents. Once internalized, AgNPs bind to thiol groups in key enzymes, inhibiting their activity and disrupting essential metabolic pathways. Simultaneously, the release of Ag⁺ ions promotes ROS generation, triggering oxidative stress that damages lipids, proteins, and nucleic acids. AgNPs also interfere with ribosomal function, thereby inhibiting protein synthesis, while their direct interaction with DNA impairs replication and transcription. Furthermore, disruption of the electron transport chain reduces ATP production, resulting in cellular energy depletion. Collectively, these mechanisms induce irreversible damage to bacterial cells, ultimately leading to cell death [84].

The bioactivity of Ag NPs is strongly influenced by their physicochemical properties, such as size, surface area, and morphology [82], which lead to varying bactericidal effects due to differences in crystal surface reactivity [85, 86]. In aqueous environments, Ag NPs undergo oxidation, releasing Ag⁺ ions that bind to thiol groups in critical cellular proteins, impairing their function and triggering ROS production. This oxidative stress compromises membrane integrity, increases permeability, and causes structural damage, ultimately leading to bacterial cell death [87–89].

The spherical morphology of AgNPs optimizes the surface area-to-volume ratio, enhancing their interaction with bacterial membranes. This structural feature facilitates penetration through bacterial cell walls, particularly in Gram-negative species, thereby increasing antibacterial efficacy. Previous studies have shown that smaller spherical nanoparticles exhibit stronger antibacterial activity than larger ones, primarily due to their greater surface area [90, 91]. The antibacterial potential of AgNPs is especially pronounced against Gram-negative bacteria, which possess a thin and permeable peptidoglycan layer that allows easier nanoparticle penetration and subsequent cell death [91, 92].

Zeta potential is a key determinant of the colloidal stability of silver nanoparticles. In biomedical applications, higher absolute values particularly negative charges indicate improved stability and reduced aggregation [92–94]. Although zeta potential measurements were not performed in this study, the synthesis method and stabilizing agents used suggest that the nanoparticles likely possess a negatively charged surface. This negative charge promotes electrostatic attraction with positively charged bacterial membranes, thereby enhancing antibacterial activity.

This study evaluated the antibacterial effects of AgI-NPs at different concentrations (1, 3, and 5 mg/mL) against four pathogenic bacteria (*E. coli* O157:H7, *S. aureus* ATCC 25923, *P. aeruginosa* ATCC 29212, and *L. monocytogenes* ATCC 7644). The antibacterial activities of AgI-NPs varied significantly among the tested pathogenic bacteria at each dose (*p <* 0.05), demonstrating differing levels of bacterial inhibition depending on the strain. The highest activity (1.70 mm) observed against *L. monocytogenes* and the lowest activity (0.50 mm) against *P. aeruginosa*. Overall, AgI-NPs synthesized from C-β-glucan isolated from *K. marxianus* M59 cell wall displayed limited antibacterial activity against all tested pathogens, as shown in Table 3, while Table 4 summarizes the antibacterial activities of Ag NPs reported in other studies for comparison.

**Table 3:** The zone diameters (mm) of AgI-NPs against pathogens

Bacteria	AgI-NPs (mg/mL)			Antibiotic Disk Ampicillin (Control)
	1 mg/mL^a^	3 mg/mL^b^	5 mg/mL^c^	
*S. aureus* ATCC 25,923^**^	0.70 ^c^	0.78	1.00 ^a^	35.10
	± 0.01	± 0.00	± 0.02	± 0.04
*L. monocytogenes* ATCC 7644^**^	0.80 ^b, c^	1.10 ^a, c^	1.70 ^a, b^	31.60
	± 0.04	± 0.02	± 0.01	± 0.05
*P. aeroginosa* ATCC 29,212^**^	0.50 ^b, c^	1.00 ^a, c^	1.30 ^a, b^	20.50
	± 0.00	± 0.01	± 0.03	± 0.02
*E. coli* O157:H7^*^	1.20	1.30	1.45	11.60
	± 0.01	± 0.01	± 0.02	± 0.03

^a, b, c^ (Difference is significant at the 0.05 level) (One-Way ANOVA-Tukey)

* Correlation is significant at the 0.05 level (Pearson correlation)

** Correlation is significant at the 0.05 level (Pearson correlation)

Values are means ± standard deviations from experiments done in triplicate

**Table 4:** Antimicrobial activity of agI-NPs and ag NPs against various patthogens

AgNP Source	Pathogens	Inhibition zone (mm)	Reference
Novel Yeast (HX-YS and LPP-12Y)	*E. coli* *S. aureus* *B. subtilis* *M. albican* *P. aeruginosa*	14.61–21.78	[86]
*Ageratum conyzoides*, *Mikania micrantha*	*B. cereus* *E. coli*	1.2–1.33	[95]
*Talinum triangulare*	*S. aureus* *E. coli* *C. albicans*	2.3, 2.55, and 1.65	[96]
*Aspergillus hiratsukae*	*E. coli* *S. aureus* *B. subtilis*	19.3, 14.9, and 22.0	[9]
*Aspergillus luchuensis*	*A. brasinsilles* *C. albicans*	47 and 42.1	[64]
*Pantoea anthophila*	*S. aureus* *S. epidermidis* *P. mirabilis* *E. coli* *S. typhi* *K. pneumoniae* *B. subtilis*	4.03–7.03	[97]
*K. marxianus* M59 Beta-Glucan	*S. aureus* *L. monocytogenes* *P. aeruginosa* *E. coli*	0.50–1.70	This study

The results of this study were observed to be similar [95, 96] or lower than those reported in other studies. This may be attributed to due to the synthesis source of nanoparticles, the tested bacteria, the isolation source, and different nanoparticles concentrations. Additionally, the synthesis of AgI-NP from beta-glucan extracted from yeast cell walls, rather than directly from the microorganism, may have contributed to the lower antimicrobial activity.

### Antibiofilm activity

Biofilms enable pathogenic microorganisms to invade hosts, adapt to their surroundings, and resist various environmental challenges. Microbial populations within biofilms exhibit reduced susceptibility to antibiotics, immune responses. Biofilms hinder the effectiveness of antimicrobial agents by creating barriers, altering microbial behavior, and harboring persistent cells. As a result, a significant focus in antimicrobial research is on developing strategies to combat biofilm formation [98, 99]. Silver nanoparticles exhibit potent anti-biofilm activity and are capable of effectively eliminating bacteria embedded within existing biofilms [100, 101].

This study investigated the antibiofilm activity of AgI-NPs at various concentrations against four pathogenic bacteria. The antibiofilm activities of AgI-NPs varied significantly among the pathogenic bacteria used for each dose (*p* < 0.05). The maximum antibiofilm activity was observed with 5 mg/mL AgI-NPs against *S. aureus* ATCC 25,923 (66.9 ± 0.5%), while the minimum activity was found with 1 mg/mL AgI-NPs against *L. monocytogenes* ATCC 7644 (15.2 ± 0.2%) (Table 5). The results demonstrated a concentration-dependent increase in biofilm inhibition for pathogens (except *S. aureus* ATCC 25923) (*p* < 0.05). These findings suggest the potential of high-concentration AgI-NPs as promising antibiofilm agents, although further research is warranted to fully elucidate their efficacy.

**Table 5:** Antibiofilm activities of AgI-NPs

Test bacteria	% Antibiofilm aktivite		
	AgI-NPs		
	1 mg/mL	3 mg/mL	5 mg/mL
*S. aureus* ATCC 25,923 ^a^	31.6 ± 0.4 ^b, d^	57.8 ± 0.2 ^b, c,d^	66.9 ± 0.5 ^b, c,d^
*L. monocytogenes* ATCC 7644 ^b, *^	15.2 ± 0.2 ^a, c^	28.0 ± 0.6 ^a, c,d^	40.1 ± 0.3 ^a, c,d^
*P. aeroginosa* ATCC 29,212 ^c, *^	29.4 ± 0.5 ^b, d^	36.5 ± 0.1 ^a, b,d^	45.4 ± 0.2 ^a, b,d^
*E. coli* O157:H7 ^d, *^	17.7 ± 0.4 ^a, c^	19.8 ± 0.2 ^a, b,c^	27.8 ± 0.7 ^a, b,c^

^a, b, c, d^ (Difference is significant at the 0.05 level) (One-Way ANOVA-Tukey)

* Correlation is significant at the 0.05 level (Pearson correlation)

Values are means ± standard deviations from experiments done in triplicate

A few studies have investigated the use of silver nanoparticles to inhibit biofilm formation. Ansari et al. [102] found that Ag NPs totally inhibited (50 µg/mL) biofilm by *E. coli* and *Klebsiella pneumoniae*. Similar findings were declared by Kalishwaralal et al. [103], who observed a 95–98% reduction in biofilm by *P. aeruginosa* and *S. epidermidis* biofilms when exposed to Ag NPs at a concentration of 100 nM. Manikandan et al. [104] studied the ability of green-synthesized silver nanoparticles to inhibit biofilm formation. They found that Ag NPs demonstrated significant antibiofilm activity against *Aeromonas hydrophila* (69%), *Pseudomonas aeruginosa* (71.37%), and *Staphylococcus aureus* (66.96%) at a concentration of 100 micrograms per milliliter. In a seperate study, AgNPs effectively inhibited biofilm formation by *B. cereus*, *K. pneumoniae*, *E. coli*, and *S. aureus* at concentrations of 50 and 100 µg/ml. Moderate inhibition was observed at 12.5 and 25 µg/ml, while no effect on biofilm formation was noted at 6.25 µg/ml for these microorganisms [65]. Yuksekdag et al. [61] found AgNPs inhibited *P. aeruginosa* and *E. coli* biofilms by 96.21% and 94.54% (40 mg/mL), and *S. epidermidis* by 68.53% (0.156 mg/mL).

Nanoparticles can effectively disrupt biofilm formation by directly interacting with cell surfaces due to their small size and high surface area [105]. Moreover, Ag NPs can modify biofilm formation and physiology by targeting key metabolic pathways, leading to reduced microbial activity and biofilm eradication [106]. The observed differences in biofilm inhibition may be attributed to a combination of factors, including the specific bacteria used in the tests, the different methods employed for synthesis, and the varying experimental conditions. Besides, it is thought that differences in synthesis sources have an important effect on biofilm inhibition.

### Antioxidant activity

Oxidative stress is characterized by an imbalance between the generation of ROS and the capacity of endogenous antioxidant defense systems, leading to oxidative modifications in biomolecules and subsequent alterations in cellular morphology and function. To counteract these effects, antioxidants serve as crucial protective agents by neutralizing free radicals and mitigating their deleterious impact on cellular integrity [84, 107, 108]. This study investigated the antioxidant properties of AgI-NPs at various concentrations and compared them to ascorbic acid. The antioxidant assays were conducted using the DPPH, superoxide anion, and hydroxyl radical scavenging methods (Table 6). The results demonstrated a concentration-dependent increase in the antioxidant activity of AgI-NPs across all three methods (*p* < 0.05). The maximum activity was seen with 5 mg/mL AgI-NPs using the DPPH method (58.9 ± 0.4%), while the lowest activity was detected with 1 mg/mL AgI-NPs using the hydroxyl radical scavenging method (31.7 ± 0.5%). Ascorbic acid, used as a control, also exhibited the highest antioxidant activity with the DPPH method (83 ± 0.3%) and the lowest activity with the hydroxyl radical scavenging method (72 ± 0.1%).

**Table 6:** Antioxidant activities of AgI-NPs

Methods	% Antioxidant activity			
	AgI-NPs			
	1 mg/mL	3 mg/mL	5 mg/mL	Ascorbic acid (1 mg/mL)
DPPH ^a, **^	40.1 ± 0.1 ^b, c^	51.2 ± 0.3 ^b, c^	58.9 ± 0.4 ^b, c^	83 ± 0.3 ^b, c^
Superoxide anion ^b, **^	36.2 ± 0.4 ^a, c^	41.8 ± 0.3 ^a, c^	50.0 ± 0.2 ^a, c^	78 ± 0.4 ^a, c^
Hydroxyl radical ^c, **^	31.7 ± 0.5 ^a, b^	38.4 ± 0.2 ^a, b^	45.2 ± 0.1 ^a, b^	72 ± 0.1 ^a, b^

^a, b, c, d^ (Difference is significant at the 0.05 level) (One-Way ANOVA-Tukey)

** Correlation is significant at the 0.05 level (Pearson correlation)

Values are means ± standard deviations from experiments done in triplicate

The antioxidant capacity of mycosynthesized Ag NPs was previously evaluated using the DPPH assay by Hikmet and Hussein [67]. The findings indicated that Ag NPs exhibited a concentration-dependent antioxidant activity, with the highest activity of 83.6% observed at a concentration of 100 µg/mL and the lowest activity of 17.0% at a concentration of 6.25 µg/mL. Abd Elghaffar et al. [64] assessed the antioxidant of Ag NPs using the DPPH (2,2-diphenyl-1-picrylhydrazyl) method. The results demonstrated that Ag NPs possess potent antioxidant properties, with their radical scavenging ability increasing from 33.0% to 85.1% as their concentration increased from 3.9 to 1.000 µg/mL. Mistry et al. [71] found that Ag NPs exhibited significantly stronger antioxidant properties (63.97%) than vitamin C (28.13%), as measured by their ability to scavenge DPPH radicals. In a seperate research, Ag NPs (0.156–40 mg/mL) exhibited scavenging efficiency 66.01–72.49% [61].

Nanoparticles with small diameters and spherical morphology tend to demonstrate superior antioxidant activity due to their enhanced physicochemical reactivity. The reduced particle size increases the surface area-to-volume ratio, thereby providing more active sites for interaction with ROS. Additionally, the uniform geometry of spherical nanoparticles facilitates consistent electron transfer dynamics, which contributes to efficient radical scavenging. These structural advantages improve the nanoparticles’ capacity to mitigate oxidative stress and protect cellular components from ROS-induced damage [109, 110]. In this study, the synthesized AgI-NPs were characterized by a spherical morphology and a size distribution ranging from 29.1 to 100.6 nm. These structural features are consistent with physicochemical profiles known to enhance antioxidant performance. Notably, increasing the concentration of AgI within the nanoparticle formulation led to a marked improvement in antioxidant capacity, demonstrating up to 58.9% radical scavenging activity. This concentration-dependent enhancement suggests that both particle morphology and AgI content synergistically contribute to the nanoparticles’ ability to scavenge free radicals and attenuate oxidative stress.

### Cytotoxicity effect of AgI-NPs

Ag NPs are increasingly recognized as potent anticancer agents due to their high surface reactivity and ability to release silver ions (Ag⁺), which trigger cytotoxic and apoptotic responses in cancer cells. The cytotoxicity of AgNPs arises primarily from their capacity to penetrate cellular membranes via endocytosis, allowing them to accumulate within intracellular compartments, particularly the mitochondria [84, 111, 112]. Once internalized, Ag NPs release Ag⁺ ions, which stimulate the generation of ROS, leading to elevated oxidative stress and widespread biochemical disruption. ROS accumulation adversely affects mitochondrial membrane potential, impairing ATP production and disrupting cellular homeostasis. This mitochondrial dysfunction plays a pivotal role in activating caspase enzymes, which are central to the intrinsic apoptotic pathway. Concurrently, ROS and Ag⁺ ions interact with intracellular biomolecules, including nucleic acids and proteins, causing denaturation, fragmentation, and irreversible damage [106, 113]. Ag⁺ ions also bind to reactive thiol groups in proteins, destabilizing cellular membranes and interfering with metabolic processes [84, 100, 114].

These combined effects mitochondrial impairment, caspase activation, and DNA damageculminate in programmed cell death. The cytotoxic effects of Ag NPs are further modulated by particle size, with smaller nanoparticles exhibiting greater toxicity due to their higher surface area and enhanced ion release capacity [61, 84, 115, 116, 117]. Moreover, the unique surface properties of metal nanoparticles, acting as redox-active systems, contribute to proinflammatory responses and adverse effects on both cancerous and healthy cells [118]. Although the precise mechanisms of Ag NP induced cytotoxicity remain under investigation, current evidence supports a multifaceted mode of action involving oxidative stress, intracellular signaling disruption, and genomic instability. In this context, the present study is among the first to explore the cytotoxicity of silver iodide (AgI) nanoparticles synthesized using yeast cell wall-derived β-glucan, offering novel insights into their biological interactions with both normal and cancer cells.

The size and morphology of nanoparticles are key physicochemical parameters influencing their cytotoxic behavior. Due to their increased surface reactivity, smaller nanoparticles tend to release ions more rapidly, intensifying intracellular stress responses. In silver-based systems, this can lead to elevated intracellular silver ion concentrations, contributing to increased oxidative stress and cellular toxicity [119, 120]. In this study, the synthesized AgI-NPs exhibited a predominantly spherical morphology with a nanoscale size distribution. This size range and shape are consistent with structural profiles known to promote cellular uptake via endocytosis, particularly in epithelial cell lines.

**Fig. 7:**
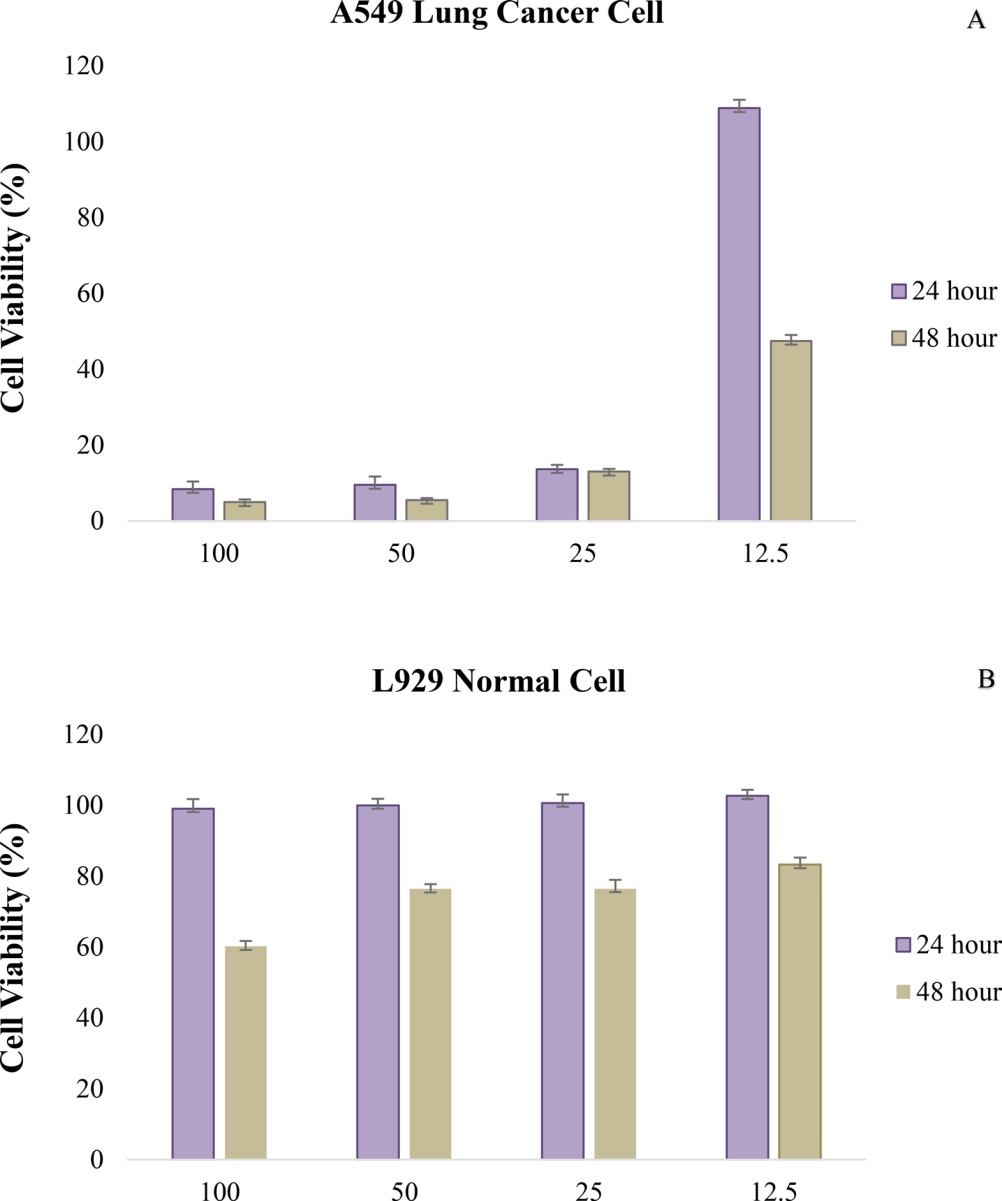
(**a**-**b**) Cytotoxicity of AgI-NP **a**) A549 lung cancer cell **b**) L929 normal cell. The error bars represent the standard deviation of the measurements

The cytotoxic effects of AgI-NPs were evaluated in L929 and A549 cell lines at 24 and 48 h using varying concentrations. A549 cells showed IC50 values of 23.95 µg/mL and 19.13 µg/mL at 24 and 48 h, respectively, indicating a time-dependent increase in sensitivity. In contrast, L929 cells exhibited higher IC50 values (130.37 µg/mL at 24 h and 73.69 µg/mL at 48 h), suggesting comparatively lower susceptibility. At 48 h, a concentration-dependent reduction in viability was observed in both cell lines. The viability of A549 cells decreased significantly with increasing concentrations, reaching 4.97% at the highest dose (100 µg/mL) (Fig. 7a). Similarly, L929 cells displayed reduced viability at 48 h, with the maximum concentration resulting in a viability of 60.23%. No apparent cytotoxic effects were detected in L929 cells at 24 h across all concentrations (Fig. 7b).

In another study, MTT assays on follicular thyroid cancer cells (FTC133) revealed an IC50 value of 95.22 µg/mL for AgI nanoparticles. Cellular uptake of AgI-NPs by FTC133 cells was found to be concentration, size, and time-dependent. Treatment with AgI NPs reduced the migration rate of FTC133 cells by 60% compared to the control group [62]. In Yuksekdag et al. [61] study, the cytotoxicity of AgNPs on L929 cell lines was evaluated after 24, 48, and 72 h at concentrations of 0.39–200 µg/mL. Low concentrations (0.39–25 µg/mL) showed no toxicity. IC50 values were 113, 117, and 162 µg/mL at 24, 48, and 72 h, respectively. Abu-Hussien et al. [65] evaluated the cytotoxicity of biogenic Ag NPs on human skin fibroblasts, demonstrating an IC50 value greater than 200 µg/mL. Hikmet and Hussein [67] conducted a study to evaluate the cytotoxicity of mycosynthesized Ag NPs against HT-29 cells. Their findings demonstrated that Ag NPs exhibited cytotoxic effect against these cancer cells, while demonstrating minimal toxicity to normal cell lines. Baker et al. [121] reported that biogenic F-Ag NPs (hypha extract of fungus) and MTX (methotrexate) exhibited dose dependent inhibition of A549 cells, with IC50 values of 21.6 and 17.7 µg/mL, respectively. F-AgNPs showed no significant toxicity against BEAS-2 cells, while MTX had an IC50 of 26 µg/mL. In another study, biosynthesized Ag NPs were found cytotoxic against the cancer cells (Caco-2). The authors stated that the green Ag NPs selectively targeted Caco-2 cells, showing an anti-proliferative effect at low concentrations. Conversely, the nanoparticles impacted the survival of normal cells only when used in high concentrations [122]. Hamouda et al. [123] found that green Ag NPs exhibited cytotoxic effects against MCF-7 and HCT-116 cancer cells, with IC50 values of 6.147 and 5.369 µg/mL, respectively.

The results of this study are generally consistent with previous findings in the literature regarding the cytotoxic effects of AgI-NPs on cancer cells. While the observed cytotoxicity of AgI-NPs aligns with data from other studies, certain differences are noted. These discrepancies may be attributed to variations in synthetic methods, nanoparticle**s** properties, and experimental conditions. Furthermore, the observed cytotoxic effects may not be exclusively attributed to silver, as the iodine ions present in AgI-NPs could also play a role in the biological activity. Iodine is known for its antimicrobial and potential anticancer properties, which may synergistically enhance the efficacy of AgI-NPs.

## Conclusion

This study presents one of the first comprehensive studies on the characterization and biological activities of silver iodide nanoparticles (AgI-NPs) synthesized via a biogenic route using β-glucan extracted from *Kluyveromyces marxianus* M59. The AgI-NPs exhibited spherical morphology (29.1–100.6 nm, mean: 52.6 ± 20.5 nm), a face-centered cubic crystalline structure, and a distinct absorption peak at 450 nm. Functionally, they demonstrated notable antibiofilm (15.2–66.9%) and antioxidant (31.7–58.9%) activities. Most importantly, the AgI-NPs showed selective cytotoxicity, with significantly lower IC50 values against A549 lung cancer cells compared to L929 normal fibroblasts, highlighting their potential as targeted anticancer agents with minimal impact on healthy cells. These findings underscore the multifunctional nature of AgI-NPs and their promise in biomedical applications, particularly in cancer therapy and biofilm-related pathologies. While the results are encouraging, the study is limited by the use of only two cell lines, which constrains the generalizability of the cytotoxicity data. Additionally, the antibacterial efficacy was lower than conventional antibiotics, and the molecular mechanisms underlying the observed biological effects remain to be elucidated. Future research should expand cytotoxicity assessments to diverse cell models and conduct mechanistic studies to explore pathways such as ROS generation and quorum sensing inhibition. Long-term in vivo evaluations will also be essential to validate the clinical potential of AgI-NPs. Despite these limitations, the current findings lay a solid foundation for the development of AgI-NP-based nanomaterials with broad biomedical relevance.

## Data Availability

The data used to support the findings of this study are included within the article.
